# The Treatment of Gingival Recessions in the Lower Anterior Region Associated with the Use/Absence of Lingual-Fixed Orthodontics Retainers: Three Case Reports Using the Laterally Closed Tunnel Technique and Parallel Incision Methods

**DOI:** 10.3390/dj13030093

**Published:** 2025-02-21

**Authors:** Alexandra Tavares Dias, Jessica Figueiredo Lopes, Juliana Campos Hasse Fernandes, Gustavo Vicentis Oliveira Fernandes

**Affiliations:** 1 Periodontics Department, State University of Rio de Janeiro (UERJ), Rio de Janeiro 20550-013, RJ, Brazil; 2 Independent Researcher, St. Louis, MO 63104, USA; 3 Missouri School of Dentistry & Oral Health, A. T. Still University, St. Louis, MO 63104, USA

**Keywords:** connective tissue, orthodontics, periodontics, case report, gingival recession

## Abstract

**Background**: The prevalence of gingival recessions (GRs) in the global population is 78%. A long-term study showed a 47% increase in the prevalence of GRs five years post-orthodontic treatment, particularly in the lower anterior region. It can be caused and/or exacerbated after orthodontic treatment, where the retainer placed can induce tooth movement or when it fails to maintain a passive position upon bonding. Thus, the goal of this case report was to present treatments for gingival recessions, with the approaches of the laterally closed tunnel technique and parallel incision methods, after orthodontic treatment in patients using non-passive lingual retainers. **Methods**: This case report adhered to the CARE guidelines. Three healthy patients were referred due to GR defects in the lower anterior region (RT1 and RT2). All patients had GR associated with deficient lingual-fixed orthodontics retainers. The same experienced periodontist (ATD) developed the surgeries and aimed to achieve root coverage using the connective tissue graft associated with a coronally advanced flap (CAF) and modify the recipient area’s gingival phenotype. **Results**: In all cases, a new orthodontic treatment was not possible due to anatomical or patient-related factors. Outcomes after six months, three years, and five years are presented, encompassing clinical and esthetic evaluations. **Conclusions**: GRs must always be addressed by orthodontic therapy or lingual-fixed orthodontic retainers. In cases where dental elements are positioned outside the bone envelope, orthodontic treatment may be considered before root coverage surgery. Therefore, surgical intervention should be undertaken for the keratinized tissue and volume gain, independently of the tooth position. Modifying the phenotype in these situations is vital for the long-term maintenance of periodontal health.

## 1. Introduction

Gingival recession (GR) is mucogingival alteration with the apical displacement of the gingival margin in relation to the cementoenamel junction (CEJ) and consequent clinical attachment loss, resulting in root surface exposure to the oral environment [1]. The etiology of GRs is multifactorial, affecting one or more teeth and involving buccal, lingual, or proximal aspects. These conditions have various clinical implications, including esthetic compromise, root caries development, non-carious cervical lesions [2], and dentin hypersensitivity [3,4]. GR prevalence in the global population is 78% [5]. GR prevalence and severity increase with age [6]; 88% of individuals over 65 years old and 50% of individuals aged 18 to 64 have more than one gingival recession [1].

**Figure 1:**
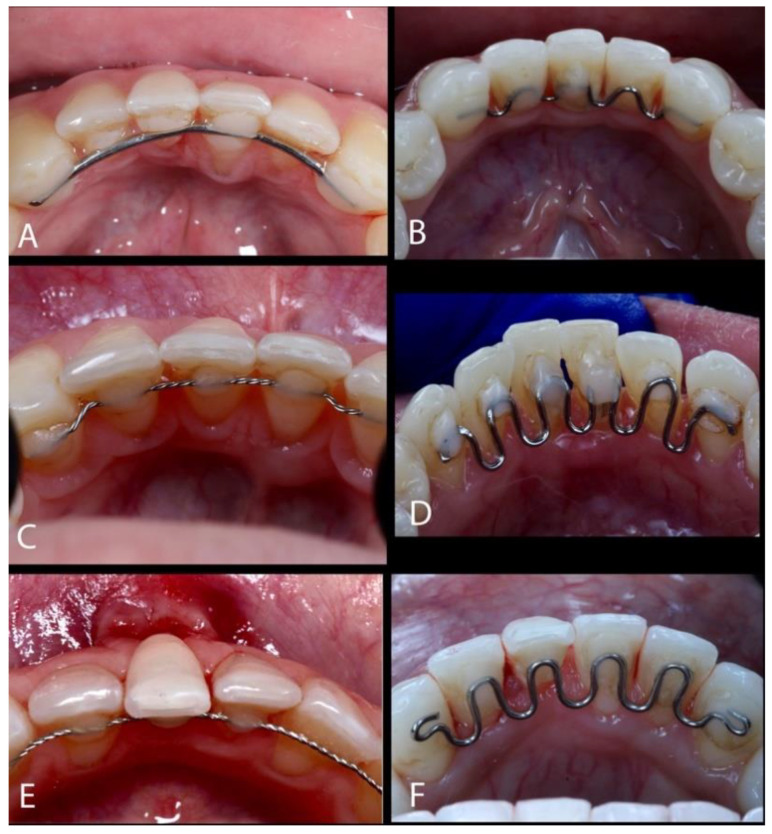
(**A**–**F**) The use of lingual-fixed orthodontic retainers.

Some predisposing factors for gingival recessions include thin periodontal phenotype, reduced alveolar bone thickness, occlusal trauma, age, traumatic tooth brushing, the use of hard-bristled toothbrushes, osseous dehiscence, and the accumulation of biofilm and calculus [7,8]. Additionally, the use of fixed orthodontic devices [9] or retainers is also a contributory factor (Figure 1A–F) [3]. Gingival recessions can be caused and/or exacerbated during or after orthodontic treatment, which can depend on the orthodontic tooth movement direction [10]. According to Djeu et al. [11], gingival recessions during orthodontic treatment range from 1.3% to 12%. Moreover, a long-term study showed an increased GR prevalence of 47% five years after orthodontic treatment [12].

Orthodontic retainers represent the final phase of orthodontic treatment. They ensure stability and maintain teeth in the achieved position at the end of treatment, preventing them from returning to their original positions [13]. Their use also aims to prevent relapse, which can occur due to the forces of periodontal fibers surrounding the teeth that tend to pull them back to their initial positions and improper occlusal contacts if the final occlusion is not ideal. Age-related changes, such as continuous dentofacial growth and changes in surrounding soft tissues, can also impact the stability of the orthodontic outcome, justifying the use of retainers [14]. Periodontal health may be compromised by some retainers due to the increased accumulation of plaque and calculus along the wire, which, if not adequately removed, increase the local inflammation and can induce adjacent hard and soft tissue loss [15].

Patients using lingual retainers should receive guidance on maintaining rigorous oral hygiene and attending regular dental visits for periodontal follow-up and retainer maintenance [15]. Intraoral retainer use is indefinite as long as periodontal health is maintained [15]. Then, regular visits to dentistry aim to detect detachment of retainer resins or any unwanted activation due to wire deformations, which can cause unwanted tooth movements and increase the risk of gingival recessions in the involved teeth [16]. The forces generated by lingual retainer wires were evaluated in vitro during simulated intrusion–extrusion and buccolingual movements [16]. It was observed that these forces could exceed 1 Ncm, sufficient to cause unwanted tooth movements during lingual retainer use. This condition can lead to gingival recession and root exposure, especially when the root position is inadequate, such as outside the bony envelope [17]. It is essential to know that the severity of these effects may vary depending on the torque differences and the degree of incisor inclination [18].

GR development or progression can occur during or after orthodontic treatment. After treatment, a continuous increase in GRs was reported by Renkema et al. [19], which was 7% at the end of treatment, 20% two years post-treatment, and 38% after 5 years. The most affected teeth were the lower incisors, followed by the upper canines, premolars, and molars [19].

Gingival recessions’ prevalence and severity follow an increase with age [6]. It seems to occur in orthodontic patients, especially those undergoing incisor proclination. Thin gingival phenotype, gingival recessions, buccal gingival thickness, and the amount of keratinized gingiva are correlated with a higher risk of GR after orthodontic treatment [20]. Furthermore, fixed retainers can produce inadvertent tooth movement, mainly when the retainer breaks but remains bonded to a tooth/teeth or when an intact retainer is not passive after fixation or distorted by function [21]. Patients might not notice partial debonding, which can create torque problems and the development of gingival recession. Therefore, observing the low number of studies and clinical cases published involving non-passive lingual retainers associated with gingival recession and the treatment performed, the aim of this case report is to demonstrate treatment of gingival recessions, using the approaches of the laterally closed tunnel technique and parallel incision methods, after orthodontic treatment in patients using non-passive lingual retainers when a new orthodontic approach is not recommended due to anatomical or patient-related factors.

## 2. Materials and Methods

This case report adhered to the CARE (Case Report) guidelines. Before any clinical procedures, the patients received information about options for treatment and signed the informed consent form. Three healthy patients arrived at the private clinic through external referral due to GR defects in the fifth sextant (lower anterior region). The gingival recessions were classified as recession type (RT) 1 and RT2. The RT1 scenario presented no interproximal clinical attachment loss; the interproximal cementoenamel junction (CEJ) was not visually detectable, and it was equivalent to Miller Class I and II. RT2 presented loss of tissue in the interproximal clinical attachment, which was less than or equal to the loss of buccal gingival attachment (recession), and it was equivalent to Miller Class III.

All patients had GR associated with deficient lingual-fixed orthodontic retainers. To establish periodontal health, microsurgeries were performed only after a pre-operative appointment and a basic periodontal approach, which included supragingival scaling and prophylaxis. The deficient retainers were replaced with new 3 × 3 lingual-fixed orthodontic retainers in all cases. The same experienced periodontist (ATD) developed the surgeries, which aimed to achieve root coverage using the connective tissue graft associated with a coronally advanced flap (CAF) and modify the recipient area’s gingival phenotype.

## 3. Results

**Figure 2:**
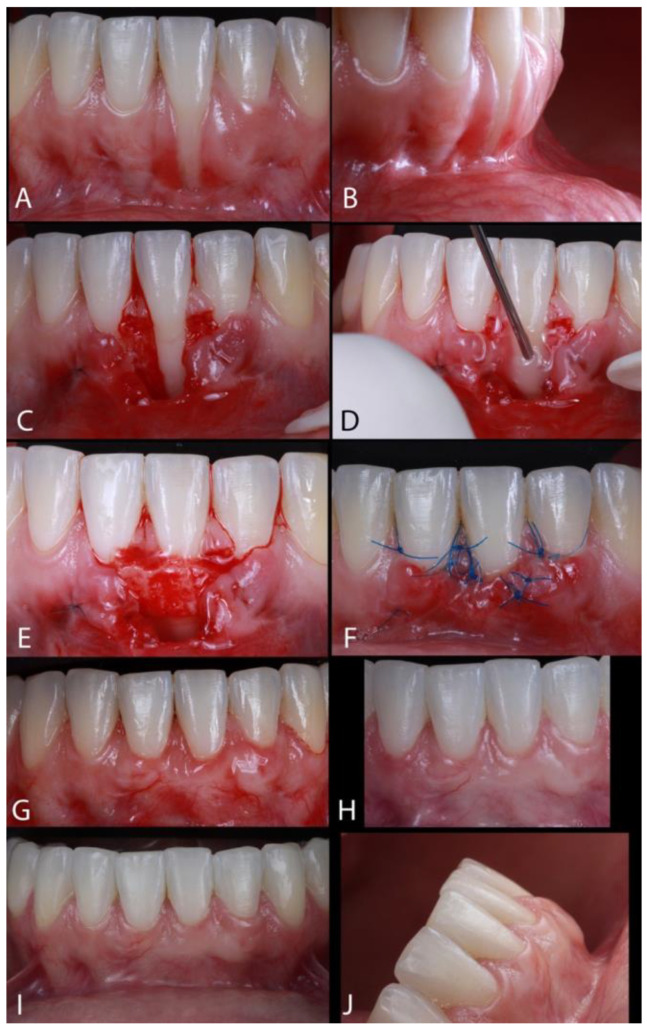
(**A**,**B**) Gingival recession type 1 (RT1). (**C**) A horizontal incision was made at the affected tooth’s mesial and distal papillae at the CEJ, and a second divergent incision extended from the CEJ of the adjacent teeth (mesial and distal) to the midpoint of the recession area. (**D**) Enamel Matrix Derivative (EMD) was applied on the root. (**E**) The insertion of the subepithelial connective tissue graft (SCTG). (**F**) The flap was sutured coronally, with polyamide 6/0 suture. (**G**) The result after 30 days. (**H**) The result after 6 months. (**I**,**J**) Follow-up after 5 years post-operative.

Case 1. A female patient, aged 32 years, had the chief complaint of “fear of losing a tooth due to GR” (Figure 2A). The patient reported no sensitivity and signs of mobility or bleeding and did not observe an increased probing depth (PD). She had completed orthodontic treatment around five years earlier; around one year after that treatment, the patient observed an increase in the buccal recession of the lower left anterior incisor. The lingual-fixed orthodontics retainer had never been replaced. The gingival recession observed in this case was classified as RT1 (Figure 2B). After applying 2% lidocaine with 1:100,000 epinephrine for local anesthesia, a horizontal incision was made at the papillae (mesial and distal) at the CEJ of the affected tooth. A second incision, diverging from the first, extended from the CEJ of the adjacent teeth (mesial and distal) to the midpoint of the recession area (Figure 2C). A 15C blade was used to perform incisions (Swann Morton^®^, Sheffield, UK); an intrasulcular incision connected those incisions. Thus, to obtain a tensionless flap, a partial-thickness flap was raised across the mucogingival junction (MGJ), permitting the gingival margin to be sutured at the first incision. The intermediary tissue (between the incisions) was removed with Castroviejo scissors (Schwert^®^, Tuttlingen, Germany), providing a uniform receptor site. The root surface exposed received PrefGel (EDTA, Ethylenediaminetetraacetic acid, 24%) (Straumann^®^, Basel, Switzerland) for 2 min, followed by saline solution irrigation. Enamel Matrix Derivative (EMD) (Emdogain, Straumann^®^, Basel, Switzerland) was used on the root (Figure 2D) before the insertion of the subepithelial connective tissue graft (SCTG) on the bed (Figure 2E). The connective tissue graft (CTG) was collected from the palate with a double-blade scalpel (Schwert^®^, Tuttlingen, Germany), with 1 mm of distance between the blades [22], on the premolar region (around 10 mm of width). Then, the epithelium was removed, and the graft positioned onto the recipient site to cover the root exposed [23]. The flaps were sutured coronally, with polyamide 6/0 suture (Resorba^®^, Hamburg, Germany) (Figure 2F). The suture of the palate was removed after 7 days, and the suture of the recipient area was removed after 14 days. The patient was seen after 7, 14, and 30 days (Figure 2G), at 6 months (Figure 2H), and at annual follow-up visits until 5 years post-operative (Figure 2I,J).

**Figure 3:**
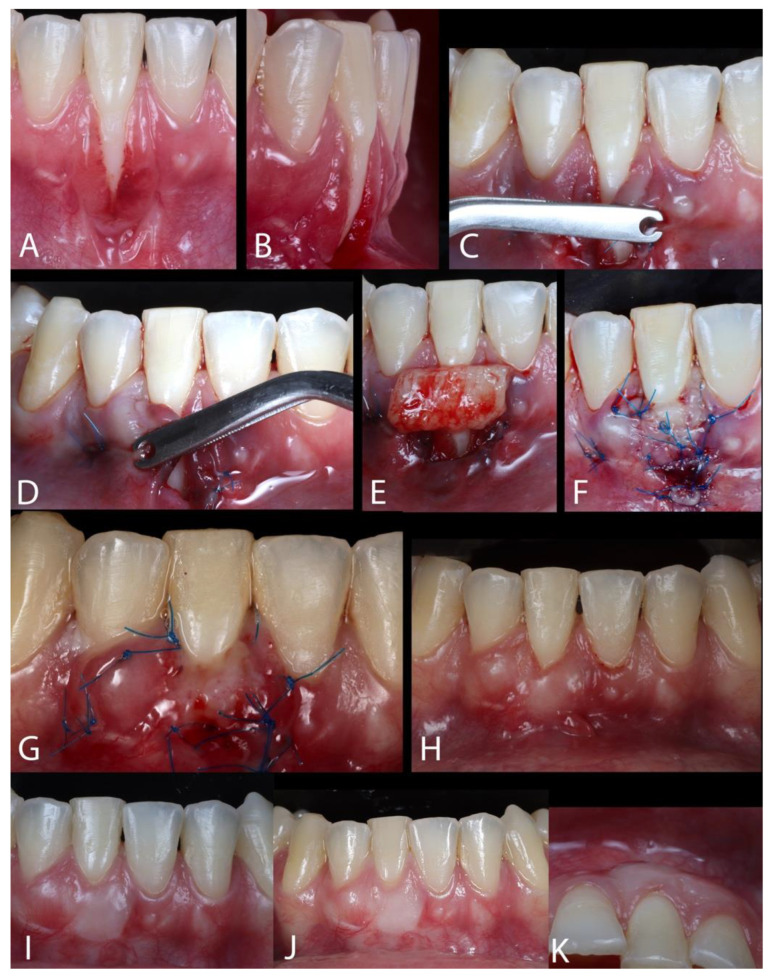
(**A**,**B**) Gingival recession type 1 (RT1). (**C**,**D**) The margins of the flap approximated without tension to cover the exposed root surface. (**E**) After removing the epithelium, the recipient site received the graft. (**F**) Interrupted sutures to accomplish the tension-free coverage of the graft and root surface. (**G**) The result after 14 days. (**H**) The result after 30 days. (**I**) The result after 18 months. (**J**,**K**) The result after 3 years.

Case 2. A 27-year-old female was referred with a chief complaint of gingival recession (Figure 3A). The patient reported no sensitivity, and signs of mobility, bleeding, or increased probing depth (PD) were detected. She had completed orthodontic treatment around 4 years earlier, but her fixed retainer broke 2 years before our first appointment. The retainer was bonded but not in all teeth. After that, the patient observed increased gingival recessions in the buccal area of the lower right anterior incisor, with lingual inclination. The retainer was changed before the surgery; the recession found was classified as RT1 (Figure 3B). After using 2% lidocaine with 1:100,000 epinephrine for local anesthesia, the laterally closed tunnel technique was used to treat the deep isolated mandibular recessions [24]. After local anesthesia, the root planing of the exposed root surface was performed with a 1–2 Gracey curette (Hu Friedy^®^, Chicago, IL, USA). Subsequently, a microsurgical blade (SB004, MJK^®^, Montpellier, France) was used to make an intrasulcular incision, and a mucoperiosteal tunnel was prepared using tunneling instruments (Helmut Zepf^®^, Seitingen-Oberflacht, Germany). The flap was prepared apically besides the mucogingival line, being extended to the mesial and distal from the recession defect until the interdental papillae’s buccal surface. Muscles and collagen fibers inserted at the apical and lateral parts, at the inner surface of the flap, were released using conventional and microsurgical blades, tunneling instruments, and curettes (Gracey) until the obtention of the tension-free (at the mesial and distal) flap, on the recession margins. Then, the flap’s margins could be moved closely without tension in order to cover entirely or significant part of the exposed root surface (Figure 3C,D). The root surface received a treatment with PrefGel (EDTA 24%) for 2 min, followed by irrigation with saline solution. After that, a palatal, 1 mm-thick SCTG was harvested on the premolar region (about 10 mm in width) using a double-blade scalpel, as described by Harris [25]. After epithelial removal, the naked root received the tissue graft that covered it (Figure 3E). Before the insertion of the connective tissue graft on the bed, EMD was applied on the root. The SCTG was stabilized to the CEJ by sling sutures with polyamide 6/0 suture, pulling together the margins of the recession over the graft; then, interrupted sutures were performed to accomplish the tension-free coverage of the graft and denuded root surface (Figure 3F). The palatal sutures were removed 7 days after surgery, whereas those from the treated teeth were removed 14 days post-operatively (Figure 3G). The patient was followed up after 7, 14, and 30 days (Figure 3H) and 6 and 18 months (Figure 3I), and annual follow-up visits were conducted until three years post-operative (Figure 3J,K). In the first follow-up, the occlusal interference was adjusted to avoid any relapse.

**Figure 4:**
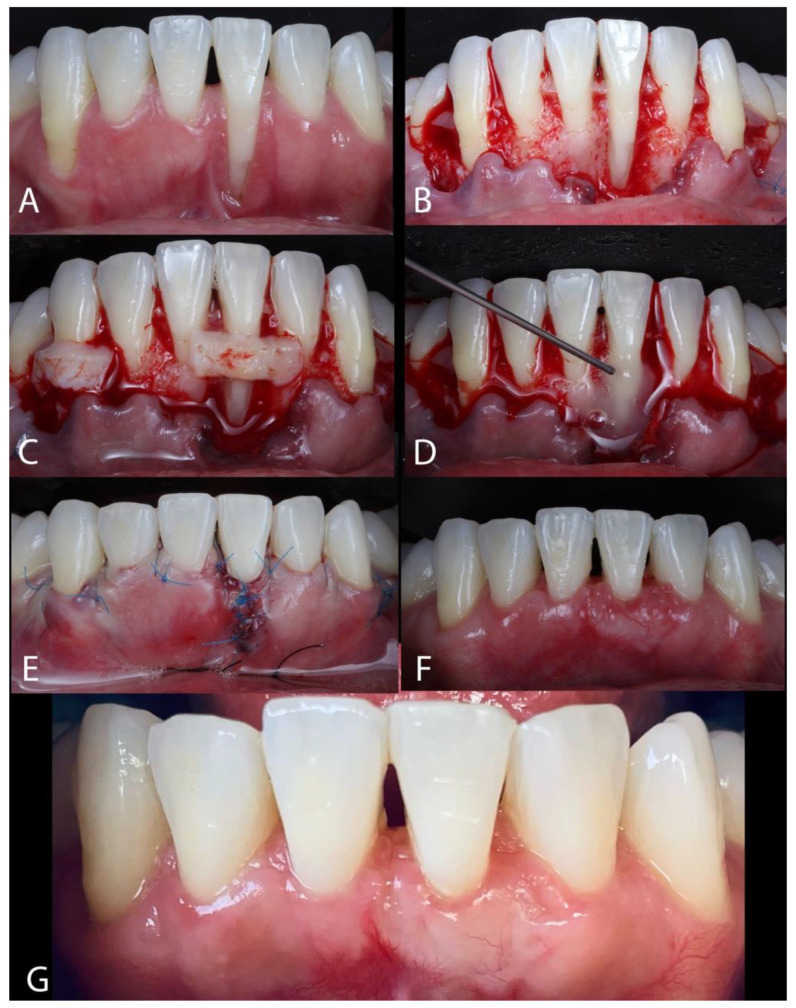
(**A**) Presence of GRs at #24 and #27. (**B**) A split-full-split flap was created, extending apically beyond the mucogingival line and extending distally to the canines. (**C**) After the epithelium was removed, the graft was placed on the recipient site, covering the exposed roots. (**D**) EMD was administered to the root. (**E**) The flap was repositioned and stabilized with sutures. (**F**) Follow-up after 30 days. (**G**) Follow-up after 6 months.

Case 3. A 23-year-old female patient was referred, presenting the primary concern of GR affecting teeth #24 and #27 (Figure 4A). The patient reported sensitivity; however, there were no observable signs of tooth mobility, bleeding, or clinically detectable increased PD. She had undergone orthodontic treatment approximately a decade prior, but her fixed retainer had become detached five years before her first appointment. Then, the patient observed increased and significant gingival recessions in the buccal area of the lower left anterior incisor and lower right canine. The recessions found were classified as RT2 and RT1 for #24 and #27, respectively. Following the administration of local anesthesia (2% lidocaine with 1:100,000 epinephrine), root planning was conducted utilizing a 1–2 Gracey curette. The horizontal incisions and flap were delineated according to Stefanini et al. [26]. A horizontal superficial incision was made at the base of the papilla using a 15C blade. Subsequently, a split-full-split flap was created, extending apically beyond the mucogingival line, severing the fibers, and extending distally until the canines (Figure 4B). PrefGel (24% EDTA) was applied to the root surface for 2 min, followed by thorough irrigation with saline solution. After that, 1 mm-thick SCTG was harvested with a double-bladed scalpel. After the removal of the epithelium, the graft was placed on the recipient site, covering the exposed roots (Figure 4C). Before the SCTG placement into the surgical bed, EMD was administered to the root (Figure 4D). The SCTG was stabilized at the CEJ with sling sutures using 6/0 polyamide sutures. The recession gingival margins were approximated over the graft and sutured using interrupted sutures to facilitate graft coverage [26]. Additionally, sutures at the deep vestibule were implemented to enhance flap stabilization (Figure 4E). The sutures placed in the palatal region were excised 7 days post-operatively, whereas those associated with the treated teeth were removed 14 days post-operatively. The patient underwent evaluations after 30 days (Figure 4F) and 6 months (Figure 4G).

Post-surgically, all patients received analgesics (400 mg Ibuprofen) twice a day for 3 days and antibiotics (500 mg Amoxicillin, three times per day, 7 days). It was recommended that the sites were not allowed to be brushed for 2 weeks post-operatively. Moreover, 0.12% chlorhexidine–digluconate mouthwash (Periogard, Colgate-Palmolive^®^, New York, NY, USA) was prescribed to be used twice daily for 1 min for 3 weeks post-surgery. Patients were instructed in how to perform the mechanical tooth cleaning of the surgical sites at 1 month post-surgery.

Following six months of treatment in cases 1 and 3 and 18 months in case 2, esthetically pleasing outcomes were attained for all the patients and the clinician despite employing distinct techniques. No additional orthodontic adjustment was performed before the surgical intervention. This follow-up indicated a positive outcome in terms of root coverage and the healing of soft tissue. Minimal or absent edema and erythema were observed during the evaluation. These results may be correlated with the patient’s administration of pre- and post-operative medications, along with the application of EMD and its potential impact on the initial phases of wound healing [23].

## 4. Discussion

The literature has pointed out the association between GRs and lingual-fixed orthodontics retainers as an iatrogenic factor [3,10,19,27]. Although there is evidence of this correlation, some short- and long-term studies have shown that fixed orthodontic retainers do not influence the increased prevalence of gingival recessions [7,13,28]. Renkema et al. [19] aimed to measure the difference in the prevalence of GRs between orthodontically treated patients using fixed orthodontic retainers 2 and 5 years after treatment. The results demonstrated an increase in the prevalence of GRs over the years, with the orthodontic treatment negatively influencing this increase. In general, some factors regarding this matter could be highlighted, such as the characteristics of the patient’s periodontal phenotype before orthodontic treatment, the type of orthodontic intervention, and the level of oral hygiene [29,30].

The gingival tissue thickness and the direction of tooth movement are crucial factors that influence changes in soft tissues during orthodontic treatment [30]. Tooth movement beyond the buccal or lingual bone cortical plate limits may trigger significant consequences for periodontal tissues [10,29]. Post-orthodontic tooth position changes can also occur due to non-passive cemented retainers, leading to increased GR. Wire activation can also occur because of the elastic deflection caused by the clinician, or as a result from masticatory forces (mechanical deformation) [28]. The long-term effectiveness of fixed mandibular cemented retainers on all six teeth (3 × 3) was evaluated over 5 years after installation [28]. Crowding was strongly related to retainer bonding failures. In six patients (2.7%), unexpected post-treatment complications (torque differences and increased buccal inclination in incisors and canines) were observed. Another publication confirmed these unexpected findings [29]. Another factor observed was the forces generated by lingual retainer wires during simulated intrusion–extrusion and buccolingual movements in vitro [17]. Forces exceeding 1 Ncm can be generated and are large enough to produce unwanted tooth movements during retainer use. Hence, GR and root exposure may occur due to a malpositioned root outside the bony envelope.

Therefore, how to treat lower buccal GRs still raises concerns; what, then, would be the first step of treatment (root coverage or orthodontic movement before any surgical procedure)? A study [31] suggested orthodontic treatment prior to surgery on root coverage in order to improve the keratinized tissue (quality and quantity) apically and laterally to the GR. Thereby, root coverage procedures will become easier, with a higher predictability for complete root coverage. Otherwise, sometimes, orthodontically repositioning a buccally displaced tooth cannot be performed for anatomical reasons. In this scenario, the technique suggested was a coronally advanced flap (CAF) with a CTG [31], as demonstrated in the cases reported. Therefore, a relatively new technique, the laterally closed tunnel technique (LCT) [24], has been used. The CAF and LCT are frequently compared due to their differing approaches and outcomes. The LCT is characterized by its minimally invasive nature, allowing for the treatment of isolated gingival recessions without the need for vertical incisions; thus, it preserves the papillae and minimizes post-operative discomfort [24,32]. Studies have reported that the LCT can achieve root coverage rates between 70% and 75%, making it a viable option for clinicians aiming for effective treatment of GR [33]. Comparing the two techniques, results have highlighted the advantages of the LCT in terms of patient comfort and esthetic outcomes. For instance, a randomized controlled trial [32] demonstrated that patients treated with the LCT reported less post-operative pain and swelling compared to those who underwent the CAF procedure. Additionally, the LCT allows for a more conservative approach, preserving the existing gingival architecture, which can be particularly beneficial in esthetic zones [24]. Conversely, while the CAF may offer higher root coverage rates, it often requires more extensive flap manipulation, which can compromise the blood supply to the tissue and potentially affect healing [34]. Furthermore, the choice of technique may also depend on the specific clinical scenario, including the type and extent of the recession. For instance, the LCT has shown promising results in treating isolated recessions, particularly in the mandibular anterior region [35]. On the other hand, the CAF remains a strong option for more extensive recession defects, where greater tissue mobilization may be necessary to achieve adequate coverage [33,36]. Ultimately, the decision between the LCT and CAF should be based on a comprehensive assessment of the patient’s specific needs, the characteristics of the gingival recession, and the clinician’s expertise with each technique.

GRs represent a significant challenge in clinical practice, and various surgical methods have been developed for root coverage, especially with the use of SCTG [37]. The success of these procedures, however, is closely related to the type of GR, which guides the choice of surgical technique and establishing the prognosis of the treatment [38]. Regardless of the type of recession, SCTG has been widely considered the gold standard, especially in situations with short or no remaining keratinized gingiva [31,37,38,39]. SCTG increases the thickness and quality of the local keratinized tissue, thereby improving the long-term stability of treatment [31]. Therefore, some discomfort can be found in the palatal healing during the initial period after surgery [40]. The correct choice of technique for harvesting and preparing the surgical bed, based on an accurate diagnosis, is the determinant for the success of the procedure and for establishing a realistic prognosis [4,39,41].

## 5. Conclusions

Gingival recessions must be addressed and treated, either by using orthodontic therapy or lingual-fixed orthodontics retainers. In cases where dental elements are positioned outside the bony envelope, orthodontic treatment may be considered before root coverage surgery. However, specific scenarios, such as the absence of a lingual bone plate, render this approach unfeasible. In such instances, surgical intervention should be undertaken, even if the positioning of the dental element is suboptimal, to facilitate the gain of keratinized tissue and enhance tissue thickness (volume). Modifying the phenotype in these situations is vital for the long-term maintenance of periodontal health. Consequently, connective tissue grafting combined with techniques for the CAF is the most recommended treatment strategy.

## Data Availability

All data are included in this article.

## Plain Language Summary

This case report delineates three cases presenting GRs and lingual retainers. Three healthy patients are included with GR, RT1 and RT2, in the fifth sextant (lower anterior region). GRs must always be addressed by orthodontic therapy or lingual-fixed orthodontic retainers. In cases where dental elements are positioned outside the bony envelope, orthodontic treatment may be considered before root coverage surgery. Therefore, surgical intervention should be undertaken for the keratinized tissue and volume gain, independently of the tooth position. Modifying the phenotype in these situations is vital for the long-term maintenance of periodontal health.
